# A rare case report of early bioprosthetic valve thrombosis presenting with acute heart failure salvaged by thrombectomy

**DOI:** 10.1186/s13019-017-0581-9

**Published:** 2017-03-27

**Authors:** Jingya Fan, Peng Teng, Yu Zou, Liang Ma

**Affiliations:** 1 Department of Cardiothoracic Surgery, The First Affiliated Hospital, School of Medicine, Zhejiang University, Hangzhou, China; 2 Postal Address: 79#, Qingchun Road, Hangzhou, 310000 Zhejiang China

**Keywords:** Bioprosthetic valve thrombosis, Aortic valve, Thrombectomy, Porcine bioprosthesis

## Abstract

**Background:**

Bioprosthetic valve thrombosis is previously considered as an extremely rare complication which hasn’t been systemically recognized and understood.

**Case presentation:**

Herein, we present an unusual case of a patient manifesting with acute heart failure, secondary to thrombus formation in a porcine aortic bioprosthesis which was implanted 11 months prior to hospitalization. Due to the patient’s poor heart function and intraoperative findings, thrombectomy was performed. For our best knowledge, cases of early bioprosthetic valve thrombosis presenting with acute heart failure have seldomly been reported.

**Conclusion:**

Our study reviews predisposing factors, typical echocardiographic features and treatment for bioprosthetic valve thrombosis and it should be considered as a reason for bioprosthetic malfunction. A combination of clinical and echocardiographic features can help for diagnosis preoperatively. In some certain circumstances, early reoperation can be avoided if anticoagulant therapy works.

## Backgound

Bioprosthetic valve is advantageous over mechanical valve as its avoidance of long-term anticoagulation and low incidence of thromboembolic events. As a result, bioprosthetic valves are being implanted with ever-increasing frequency. However, according to previous study, the reported incidence of bioprosthetic valve thrombosis (BPVT) on routine echocardiographic surveillance is approximately 6% [1]. Thus, once anticoagulant treatment has been withdrawn, BPVT should be considered as a potential cause of prosthetic malfunction. Here we present a rare case requiring early reoperation for BPVT on aortic position which can provide us a better understanding of BPVT.

## Case presentation

A 61-year old Chinese male presented with recurrent chest discomfort on exertion for 3 months and one episode of presyncope on exertion one day before referred to our hospital. 11 months ago, the patient had received aortic valve replacement, because of congenital bicuspid aortic valve, with a 21-mm Hancock II porcine bioprosthesis (Medtronic, Inc, Minneapolis, MN, USA) implanted following the protocol provided by the manufacturer. Postoperative transthoracic echocardiography (TTE) showed normal function of bioprosthesis and he made an uneventful postoperative hospital course. The patient was discharged on 10th postoperative day and was on anticoagulation therapy with warfarin (target international normalized ratio (INR): 1.8-2.5) for 6 months. He continued to follow up at our hospital postoperatively.

**Fig. 1:**
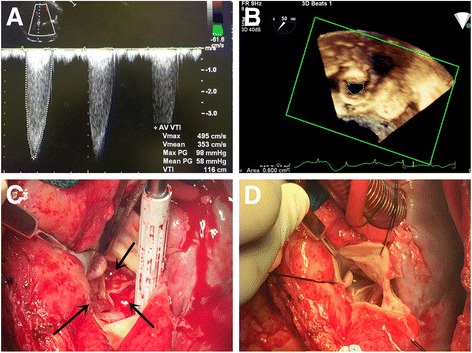
**a**, **b** The TEE detected an increased mean transvalvular gradient of 58 mmHg, a peak velocity of 4.95 m/sec and a restricted bioprosthesis opening of about 0.6 cm^2^. **c** The intraoperative view of the bioprosthetic valve, with massive thrombus (*in black arrow*) involving all the leaflets. **d** The bioprosthetic valve after thrombectomy showed smooth surface and normal function. (*TEE* transesophageal echocardiography)

After the patient’s emergent hospitalization, full laboratory workups and imaging tests were performed. Physical examination detected tachycardia, a III grade systolic murmur in the aortic valve area and diffuse moist rales in both lungs. Transesophageal echocardiography (TEE) detected a severe aortic bioprosthesis stenosis, with a mean transvalvular gradient of 58 mmHg, a peak velocity of 4.95 m/sec (Fig. 1a), and an aortic valve area of about 0.6 cm^2^ (Fig. 1b). Left ventricular function had also deteriorated, compared with previous TTE 9 months ago, with an ejection fraction of 45%. The coagulation function test showed the INR in the target range. The other tests, including complete blood cells, blood chemistry, cardiac enzymes and blood gas analysis, were unremarkable. Because of poor recognition of BPVT, early bioprosthetic valve failure was considered as the major reason for the increased transvalvular gradient across the aortic bioprosthesis. However, the patient remained in significant hemodynamic instability despite of intravenous application of diuretics and inotropics. Due to his progressive decompensated heart function, a decision of emergent salvage procedure was made after comprehensive consideration.

Intraoperatively, regular cardiopulmonary bypass (CPB) was established after re-sternotomy. Surgical exploration of the aortic bioprosthesis revealed thrombosis of the three cusps on the aortic side, limiting the opening of the valve. In addition, we noticed that the thrombus uniformly and loosely adhered to the aortic side of all the leaflets inducing diffuse valvular thickening and resulting in restricted leaflet motion and stenosis (Fig. 1c). In consideration of patient’s poor heart function and loosely adhesion of thrombus, thrombectomy, compared with redo valve replacement, seemed to be the better choice which can provide shorter aortic cross-clamp time and CPB time, less ischemic myocardial damage and a favorable short-term prognosis. The thrombectomy was successfully performed (Fig. 1d) and the CPB weaning was uneventful. Finally, the aortic cross-clamp time was 25 min and the CPB time was 41 min.

The postoperative microbiological investigation was negative and the histopathological analysis demonstrated thrombus composed mainly of platelets, erythrocytes and fibrin. The patient was discharged on warfarin with a target INR of 2–2.5 for 6 months and was followed up for over 3 years without transvalvular gradient increasing.

## Discussion

Bioprosthetic valves remain a very appealing option for patients receiving valve replacement because of the avoidance of long-term anticoagulation. Clinically, significant BPVT is considered extremely uncommon. However, in an echocardiographic surveillance study, Oliver et al. found BPVT in 10 of 161 patients (6.2%) with evidence of bioprosthesis dysfunction suggesting that BPVT has a higher incidence than was previously thought. In addition, Egbe et al. identified BPVT in 46 out of 397 (11%) bioprosthetic valves explanted at Mayo Clinic, which also suggested BPVT a more frequent cause of bioprosthesis dysfunction than was thought. In contrast to the misconception that BPVT is a perioperative phenomenon, Egbe’s study also revealed that 65% of all reoperations because of BPVT occurred more than 1 year after implantation, and up to 15% of these reoperations occurred more than 5 years [2]. Moreover, a previous study by Brown et al. identified 8 cases of BPVT in porcine valves in a 16-year retrospective research, none of which occurred in pericardial valves [3]. Egbe’s study supported this phenomenon that porcine valves were the most common type in BPVT, accounting for 36 of the 46 cases (78%), while pericardial valves accounted for 8 of the 46 cases (18%). However, the mechanism of the different occurrence of BPVT between these types of valves remains unclear.

Based on previous study, BPVT is generally associated with predisposing factors such as low cardiac output, left atrial dilatation, prior history of thromboembolic events, atrial fibrillation and hypercoagulability. In this case, however, there was a lack of apparent precipitating factors for thrombosis, as the patient had no underlying coagulation abnormality, left atrial dilatation or atrial fibrillation. As for low cardiac output, the patient did present corresponding signs such as decreased tolerance on exertion, decreased ejection fraction of 45% on TEE and dependence on diuretics and inotropics to maintain hemodynamic stability. But in our opinion, the low cardiac output in this case was much more probable a clinical manifestation of acute heart failure secondary to BPVT because all the workups and imaging exams were unremarkable during his follow-up. In addition, it can’t be excluded that BPVT could be a consequence of the episode of paroxysmal atrial fibrillation or a transient bacterial endocarditis.

Preoperative diagnosis of BPVT is not easy which need take all the clinical factors, such as clinical presentation, laboratory workups and imaging exams, into consideration. BPVT usually manifests as chronic progressive dysfunction detected on follow-up echocardiography in asymptomatic or mildly symptomatic patients. When hemodynamics is further compromised, patient can present with myocardial infarction, pulmonary edema, even cardiogenic shock and so on. Egbe et al. have proposed a BPVT diagnostic model based on echocardiographic characteristics, which are a 50% increase in prosthetic gradient within 5 years of implantation, increased cusp thickness, and abnormal cusp mobility. The presence of all three echocardiographic features reliably diagnosed BPVT with a sensitivity of 72% and a specificity of 90% [2].

The optimal treatment of the BPVT deserves further comment. Although, thrombolytic therapy is an established first-line therapy for high-risk patients with mechanical valve thrombosis [4, 5], the optimal treatment of BPVT is still a matter of debate. Anticoagulation and operative intervention is the mainstay of treatment. Operative intervention, including redo valve replacement and thrombectomy, can provide an immediate hemodynamic improvement and symptom remission. Redo valve replacement is generally considered with favorable long-term outcome while it may be associated with a higher risk of perivalvular leakage and a longer CPB time. However, thrombectomy, if it can be easily performed, usually comes out with a lower risk of perivalvular leakage and a shorter CPB time while its long-term outcome remains unclear. In our opinion, redo valve replacement is suggested in patients with compensated heart function while thrombectomy is suggested if the thrombus can be easily removed, especially in patients with decompensated heart function. Anticoagulant treatment with unfractionated heparin, which had been reported effective by other studies, may provide an early complete resolution of the process [6], which can reduce the pain and cost brought from surgery. A response to anticoagulation therapy for BPVT is usually observed within 4–12 weeks after initiation. However, the optimal duration for trial of anticoagulation therapy and the most effective anticoagulation (vitamin K antagonist vs. novel oral anticoagulant) remain unknown. In our case, given the patient’s hemodynamic instability, we thought the patient need early operative intervention and the intraoperative aortic cross-clamp time and CPB time should be as short as possible which can provide a favorable short-term prognosis. Thus, thrombectomy was finally performed.

## Conclusion

BPVT is an under recognized complication than was previously thought. It may lead to rapid bioprosthesis dysfunction. The diagnosis of BPVT remains very challenging due to a general lack of awareness of its existence. A combination of clinical and echocardiographic features is helpful for diagnosis. It is crucial and worthy to consider the diagnosis of BPVT before referring for reoperation because anticoagulant therapy may work and reverse the bioprosthesis dysfunction.

## Abbreviations

BPVT: Bioprosthetic valve thrombosis
CPB: Cardiopulmonary bypass
INR: International normalized ratio
TEE: Transesophageal echocardiography
TTE: Transthoracic echocardiography

## References

[1] Oliver JM, Galloge P, Gonzalez A, Dominguez FJ, Gamallo C, Mesa JM (1996). Bioprosthetic mitral valve thrombosis: clinical profile, Transesophageal echocardiographic features, and follow-up after anticoagulant therapy. J Am Soc Echocardiogr.

[2] Egbe AC, Pislaru SV, Pellikka PA, Poterucha JT, Schaff HV, Maleszewski JJ (2015). Bioprosthetic valve thrombosis versus structural failure: clinical and echocardiographic predictors. J Am Coll Cardiol.

[3] Brown ML, Park SJ, Sundt TM, Schaff HV (2012). Early thrombosis risk in patients with biologic valves in the aortic position. J Thorac Cardiovasc Surg.

[4] Manteiga R, Carlos Souto J, Altes A (1998). Short-course thrombolysis as the first line therapy for cardiac valve thrombosis. J Thorac Cardiovasc Surg.

[5] Lengyel M, Fuster V, Keltai M, Roudaut R, Schulte HD, Seward JB (1997). Guidelines for management of left-sided prosthetic valve thrombosis: a role for thrombolytic therapy. Consensus conference on prosthetic valve thrombosis. J Am Coll Cardiol.

[6] Pislaru SV, Hussain I, Pellikka PA, Maleszewski JJ, Hanna RD, Schaff HV (2015). Misconceptions, diagnostic challenges and treatment opportunities in bioprosthetic valve thrombosis: lessons from a case series. Eur J Cardiothoracic Surg.

